# *N*-(2,6-Dimeth­oxy­pyridin-3-yl)-9-methyl-9*H*-carbazole-3-sulfonamide

**DOI:** 10.1107/S1600536813007460

**Published:** 2013-03-23

**Authors:** Guangzhi Shan, Zhuorong Li, Laixing Hu, Jiandong Jiang, Zongying Liu

**Affiliations:** a Institute of Medicinal Biotechnology, Chinese Academy of Medical Sciences and Peking Union Medical College, Tiantan Xili 1#, Beijing, People’s Republic of China

## Abstract

In the title compound, C_20_H_19_N_3_O_4_S, a novel tubulin ligand active against human cancer, the dihedral angle between the pyridine ring and the carbazole ring system is 42.87 (10)°. In the crystal, the mol­ecules are held together by N—H⋯O and C—H⋯O hydrogen bonds into layers, which are assembled into a three-dimensional network *via* π–π stacking inter­actions between inversion-related pyridine rings, with centroid–centroid distances of 3.5101 (12) Å.

## Related literature

For the synthesis and properties of the compound and its derivatives, see Hu *et al.* (2007 ▶). For tubulin as a target for anti­cancer activity, see Wang *et al.* (2008 ▶); Jackson *et al.* (2007 ▶); Jordan *et al.* (1991 ▶); Mollinedo & Gajate (2003 ▶); Wilson *et al.* (1999 ▶); Yvon *et al.* (1999 ▶). For the stability of the temperature controller used for the data collection, see Cosier & Glazer (1986 ▶).
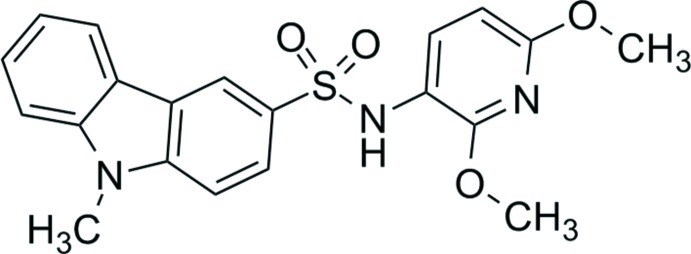


## Experimental

### 

#### Crystal data

C_20_H_19_N_3_O_4_S
*M* *_r_* = 397.45
Monoclinic, 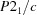

*a* = 13.5078 (2) Å
*b* = 7.9272 (1) Å
*c* = 20.9276 (3) Å
β = 124.027 (1)°
*V* = 1857.20 (4) Å^3^
*Z* = 4
Cu *K*α radiation
μ = 1.83 mm^−1^
*T* = 120 K
0.45 × 0.36 × 0.32 mm

#### Data collection

Agilent Xcalibur (Atlas, Gemini ultra) diffractometer
Absorption correction: multi-scan (*CrysAlis PRO*; Agilent, 2011 ▶) *T*_min_ = 0.475, *T*_max_ = 0.556
11460 measured reflections
3280 independent reflections
3161 reflections with *I* > 2σ(*I*)
*R*_int_ = 0.027

#### Refinement

*R*[*F* ^2^ > 2σ(*F* ^2^)] = 0.034
*wR*(*F* ^2^) = 0.088
*S* = 1.06
3280 reflections
262 parameters
H atoms treated by a mixture of independent and constrained refinement
Δρ_max_ = 0.45 e Å^−3^
Δρ_min_ = −0.52 e Å^−3^

### 

Data collection: *CrysAlis PRO* (Agilent, 2011 ▶); cell refinement: *CrysAlis PRO*; data reduction: *CrysAlis PRO*; program(s) used to solve structure: *SHELXS97* (Sheldrick, 2008 ▶); program(s) used to refine structure: *SHELXL97* (Sheldrick, 2008 ▶); molecular graphics: *SHELXTL* (Sheldrick, 2008 ▶); software used to prepare material for publication: *SHELXTL*, *PLATON* (Spek, 2009 ▶) and *publCIF* (Westrip, 2010 ▶).

## Supplementary Material

Crystal structure: contains datablock(s) I, global. DOI: 10.1107/S1600536813007460/pk2469sup1.cif

Structure factors: contains datablock(s) I. DOI: 10.1107/S1600536813007460/pk2469Isup2.hkl

Supplementary material file. DOI: 10.1107/S1600536813007460/pk2469Isup3.cml

Additional supplementary materials:  crystallographic information; 3D view; checkCIF report

## Figures and Tables

**Table 1:** Hydrogen-bond geometry (Å, °)

*D*—H⋯*A*	*D*—H	H⋯*A*	*D*⋯*A*	*D*—H⋯*A*
N1—H1⋯O3^i^	0.81 (2)	2.56 (2)	3.3387 (18)	163 (2)
C18—H18*A*⋯O1^ii^	0.96	2.45	3.398 (2)	170
C10—H10⋯O2^iii^	0.93	2.56	3.4887 (19)	177

Symmetry codes: (i) 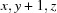; (ii) 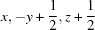; (iii) 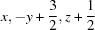.

**Fig. 1.**
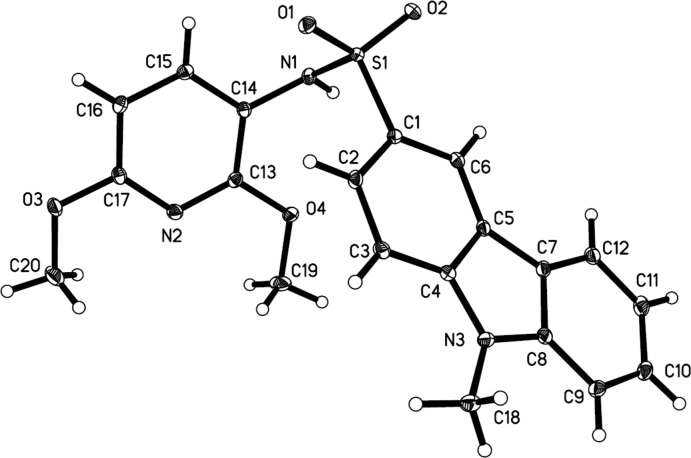
The molecular structure of title compound, showing the atom-numbering scheme. The displacement parameters are shown at the 30% probability level.

**Fig. 2.**
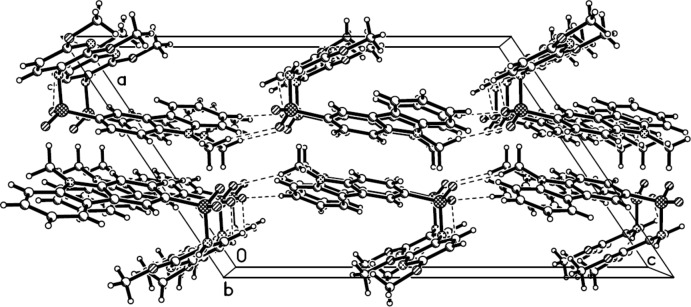
Part of packing of the title compound, viewed down the b direction. Dashed lines indicate hydrogen bonds.

## supplementary crystallographic information

### Comment

Tubulin is a target for anticancer drugs (Jordan *et al.*, 1991; Yvon *et al.*, 1999; Wilson *et al.*, 1999). The representative with this mode of action are the Vinca alkaloids (such as vincristine) and Taxol analogues such as paclitaxel (Jackson *et al.*, 2007; Mollinedo *et al.*, 2003). In this research area, *N*-(2,6-dimethoxypyridine-3-yl)-9-methylcarbazole-3-sulfonamide (IG-105, IMB-105) showed a promising anti-proliferative activity in human cancer cell lines. The title compound inhibits micro-tubule assembly by binding at the colchicine pocket and shows a potent anticancer activity *in vitro* and *in vivo* and was safe in mice (Wang *et al.*, 2008).

The title molecule is shown in Fig. 1. In the crystal structure, the carbazole CH_3_ hydrogens are disordered. The distance is 5.0286 (12) Å between the respective centroids of pyridine ring and the 6-membered ring C1\C2\C3\C4\C5\C6, and the dihedral angle between their planes is 42.87 (10) °. The intermolecular interactions that are present in the structure are N—H···O and C—H···O hydrogen bonds (Table 1) and π-π stacking interactions between inversion-related pyridine rings, with centroid-centroid distance = 3.5101 (12) Å (symmetry codes *x*, *y*, *z* and 2*-x*, *-y*, 1*-z*).

### Experimental

To a solution of 3-amino-2,6-dimethoxypyridine (2.6 g, 16.8 mmol) in 45 ml dimethylfornamide at room temperature, prepared 9-methylcarbazole-3-sulfonyl chloride (5.0 g, 16.9 mmol) was added. After stirring for 5 min, triethylamine (3.6 ml, 25.6 mmol) was added, with continued stirring for 2 h. After adding ice water (50 ml), the precipitate was filtered, washed with water (20 ml) and dried, recrystallized with anhydrous ethanol, dried *in vacuo* to give *N*- (2,6-dimethoxypyridine-3-yl)-9-methylcarbazole-3-sulfonamide as a colourless crystalline solid (5.2 g, 78%; mp: 170–172 °C).

^1^H NMR (DMSO δ): 3.40 (3*H*, s), 3.69 (3*H*, s), 3.89 (3*H*, s), 6.28(1*H*, d, *J* = 8.0 Hz), 7.26 (1*H*, t, *J* = 7.2 Hz), 7.44 (1*H*, d, *J* = 8.0 Hz), 7.52(1*H*, dd, *J* = 8.0, 7.2 Hz), 7.63 (1*H*, d, *J* = 8.0 Hz), 7.70 (1*H*, d, *J* = 8.8 Hz), 7.76 (1*H*, d, *J* = 8.8 Hz), 8.21 (1*H*, d, *J* = 8.0 Hz), 8.49 (1*H*, s), 9.32 (1*H*, s).

^13^C NMR (DMSO δ): 160.2, 156.7, 142.2, 141.3, 139.3, 130.4, 126.7, 124.3, 121.6, 121.1, 120.5, 119.9, 119.8, 112.2, 109.8, 109.1, 100.6, 53.4, 52.9, 29.2.

Single crystals suitable for X-ray analysis were obtained by slow evaporation of a mixed solvent of dichloromethane and cyclohexane (3:1 *v*/*v*).

### Refinement

All H-atoms bound to carbon were refined using a riding model with d(C—H) = 0.93–0.96 Å, *U*iso(H) = 1.2*U*eq(C) or 1.5 *U*eq(methyl C). Hydrogen atoms bonded to nitrogen atoms (N1) were located in a difference map and their positions refined using fixed isotropic U values.

### Crystal data

**Table:**

C_20_H_19_N_3_O_4_S	*F*(000) = 832
*M**_r_* = 397.45	*D*_x_ = 1.421 Mg m^−^^3^
Monoclinic, *P*2_1_/*c*	Melting point = 443–445 K
Hall symbol: -P 2ybc	Cu *K*α radiation, λ = 1.54184 Å
*a* = 13.5078 (2) Å	Cell parameters from 7976 reflections
*b* = 7.9272 (1) Å	θ = 3.3–66.7°
*c* = 20.9276 (3) Å	µ = 1.83 mm^−^^1^
β = 124.027 (1)°	*T* = 120 K
*V* = 1857.20 (4) Å^3^	Block, colourless
*Z* = 4	0.45 × 0.36 × 0.32 mm

### Data collection

**Table:**

Agilent Xcalibur (Atlas, Gemini ultra) diffractometer	3280 independent reflections
Radiation source: Enhance Ultra (Cu) X-ray Source	3161 reflections with *I* > 2σ(*I*)
Mirror monochromator	*R*_int_ = 0.027
Detector resolution: 10.4713 pixels mm^-1^	θ_max_ = 66.8°, θ_min_ = 4.0°
ω scans	*h* = −16→13
Absorption correction: multi-scan (*CrysAlis PRO*; Agilent, 2011)	*k* = −9→8
*T*_min_ = 0.475, *T*_max_ = 0.556	*l* = −24→24
11460 measured reflections	

### Refinement

**Table:**

Refinement on *F*^2^	Secondary atom site location: difference Fourier map
Least-squares matrix: full	Hydrogen site location: inferred from neighbouring sites
*R*[*F*^2^ > 2σ(*F*^2^)] = 0.034	H atoms treated by a mixture of independent and constrained refinement
*wR*(*F*^2^) = 0.088	*w* = 1/[σ^2^(*F*_o_^2^) + (0.0468*P*)^2^ + 1.1436*P*] where *P* = (*F*_o_^2^ + 2*F*_c_^2^)/3
*S* = 1.06	(Δ/σ)_max_ < 0.001
3280 reflections	Δρ_max_ = 0.45 e Å^−^^3^
262 parameters	Δρ_min_ = −0.52 e Å^−^^3^
0 restraints	Extinction correction: *SHELXL97* (Sheldrick, 2008), Fc^*^=kFc[1+0.001xFc^2^λ^3^/sin(2θ)]^-1/4^
Primary atom site location: structure-invariant direct methods	Extinction coefficient: 0.0078 (4)

### Special details

**Table:**

Experimental. The crystal was placed in the cold stream of an Oxford Cryosystems open-flow nitrogen cryostat (Cosier & Glazer, 1986) with a nominal stability of 0.1 K.
Geometry. All esds (except the esd in the dihedral angle between two l.s. planes) are estimated using the full covariance matrix. The cell esds are taken into account individually in the estimation of esds in distances, angles and torsion angles; correlations between esds in cell parameters are only used when they are defined by crystal symmetry. An approximate (isotropic) treatment of cell esds is used for estimating esds involving l.s. planes.
Refinement. Refinement of F^2^ against ALL reflections. The weighted R-factor wR and goodness of fit S are based on F^2^, conventional R-factors R are based on F, with F set to zero for negative F^2^. The threshold expression of F^2^ > 2sigma(F^2^) is used only for calculating R-factors(gt) etc. and is not relevant to the choice of reflections for refinement. R-factors based on F^2^ are statistically about twice as large as those based on F, and R- factors based on ALL data will be even larger.

### Fractional atomic coordinates and isotropic or equivalent isotropic displacement parameters (Å^2^)

**Table:**

	*x*	*y*	*z*	*U*_iso_*/*U*_eq_	Occ. (<1)
S1	0.69096 (3)	0.28850 (4)	0.403260 (19)	0.01331 (14)	
O1	0.62355 (9)	0.15207 (14)	0.35261 (6)	0.0178 (3)	
O2	0.68743 (10)	0.45199 (14)	0.37286 (6)	0.0195 (3)	
O3	0.95214 (10)	−0.42247 (14)	0.55767 (6)	0.0209 (3)	
O4	0.93551 (10)	0.14336 (14)	0.60496 (6)	0.0208 (3)	
N1	0.83268 (12)	0.22943 (17)	0.45142 (8)	0.0152 (3)	
H1	0.8755 (17)	0.301 (3)	0.4817 (11)	0.019 (5)*	
N2	0.94314 (11)	−0.14034 (16)	0.58206 (7)	0.0166 (3)	
N3	0.59643 (11)	0.36785 (16)	0.64261 (7)	0.0167 (3)	
C1	0.65608 (13)	0.30951 (19)	0.47199 (8)	0.0137 (3)	
C2	0.61415 (13)	0.16778 (19)	0.48958 (9)	0.0161 (3)	
H2	0.6013	0.0675	0.4629	0.019*	
C3	0.59167 (13)	0.1756 (2)	0.54624 (9)	0.0169 (3)	
H3	0.5632	0.0822	0.5581	0.020*	
C4	0.61299 (13)	0.32797 (19)	0.58516 (8)	0.0148 (3)	
C5	0.65942 (13)	0.46985 (18)	0.56913 (8)	0.0141 (3)	
C6	0.67969 (13)	0.46111 (19)	0.51119 (8)	0.0143 (3)	
H6	0.7082	0.5539	0.4990	0.017*	
C7	0.67359 (13)	0.6016 (2)	0.62161 (8)	0.0158 (3)	
C8	0.63249 (13)	0.5339 (2)	0.66488 (8)	0.0163 (3)	
C9	0.63143 (14)	0.6286 (2)	0.72079 (9)	0.0203 (3)	
H9	0.6032	0.5837	0.7488	0.024*	
C10	0.67411 (15)	0.7918 (2)	0.73269 (9)	0.0224 (4)	
H10	0.6743	0.8583	0.7694	0.027*	
C11	0.71695 (15)	0.8595 (2)	0.69094 (9)	0.0219 (4)	
H11	0.7462	0.9693	0.7009	0.026*	
C12	0.71658 (14)	0.7660 (2)	0.63491 (9)	0.0188 (3)	
H12	0.7444	0.8121	0.6069	0.023*	
C13	0.91249 (13)	0.01614 (19)	0.55524 (9)	0.0150 (3)	
C14	0.86006 (13)	0.05938 (19)	0.47790 (8)	0.0141 (3)	
C15	0.83742 (13)	−0.0704 (2)	0.42735 (9)	0.0160 (3)	
H15	0.8028	−0.0469	0.3754	0.019*	
C16	0.86582 (13)	−0.2349 (2)	0.45332 (9)	0.0173 (3)	
H16	0.8493	−0.3239	0.4198	0.021*	
C17	0.92017 (13)	−0.26123 (19)	0.53180 (9)	0.0163 (3)	
C18	0.54002 (16)	0.2605 (2)	0.66988 (10)	0.0239 (4)	
H18A	0.5740	0.2834	0.7234	0.036*	0.61 (2)
H18B	0.5530	0.1443	0.6637	0.036*	0.61 (2)
H18C	0.4558	0.2831	0.6405	0.036*	0.61 (2)
H18D	0.4812	0.1905	0.6284	0.036*	0.39 (2)
H18E	0.5022	0.3296	0.6880	0.036*	0.39 (2)
H18F	0.5994	0.1908	0.7112	0.036*	0.39 (2)
C19	0.99079 (19)	0.0978 (2)	0.68440 (10)	0.0319 (4)	
H19A	0.9996	0.1965	0.7137	0.048*	
H19B	1.0680	0.0497	0.7041	0.048*	
H19C	0.9418	0.0167	0.6885	0.048*	
C20	1.01784 (18)	−0.4455 (2)	0.63937 (10)	0.0326 (4)	
H20A	1.0864	−0.3728	0.6641	0.049*	
H20B	1.0434	−0.5608	0.6518	0.049*	
H20C	0.9678	−0.4185	0.6571	0.049*	

### Atomic displacement parameters (Å^2^)

**Table:**

	*U*^11^	*U*^22^	*U*^33^	*U*^12^	*U*^13^	*U*^23^
S1	0.0165 (2)	0.0117 (2)	0.0116 (2)	0.00097 (13)	0.00782 (16)	0.00084 (13)
O1	0.0184 (5)	0.0184 (6)	0.0143 (5)	0.0000 (4)	0.0077 (4)	−0.0026 (4)
O2	0.0266 (6)	0.0147 (6)	0.0192 (6)	0.0027 (4)	0.0141 (5)	0.0049 (4)
O3	0.0268 (6)	0.0119 (5)	0.0196 (6)	0.0000 (4)	0.0103 (5)	0.0013 (4)
O4	0.0320 (6)	0.0150 (6)	0.0142 (5)	−0.0026 (5)	0.0123 (5)	−0.0031 (4)
N1	0.0163 (7)	0.0117 (7)	0.0168 (7)	−0.0023 (5)	0.0088 (6)	−0.0022 (5)
N2	0.0185 (6)	0.0149 (7)	0.0160 (6)	−0.0023 (5)	0.0094 (5)	−0.0001 (5)
N3	0.0223 (7)	0.0151 (7)	0.0159 (6)	−0.0003 (5)	0.0128 (6)	0.0003 (5)
C1	0.0148 (7)	0.0133 (7)	0.0118 (7)	0.0011 (6)	0.0067 (6)	0.0000 (6)
C2	0.0188 (7)	0.0107 (7)	0.0180 (7)	−0.0013 (6)	0.0098 (6)	−0.0025 (6)
C3	0.0207 (8)	0.0121 (7)	0.0193 (8)	−0.0025 (6)	0.0121 (7)	0.0009 (6)
C4	0.0160 (7)	0.0144 (7)	0.0134 (7)	0.0018 (6)	0.0078 (6)	0.0018 (6)
C5	0.0152 (7)	0.0108 (7)	0.0134 (7)	0.0010 (6)	0.0062 (6)	−0.0003 (6)
C6	0.0152 (7)	0.0115 (7)	0.0155 (7)	−0.0001 (6)	0.0081 (6)	0.0014 (6)
C7	0.0161 (7)	0.0145 (7)	0.0134 (7)	0.0028 (6)	0.0062 (6)	−0.0006 (6)
C8	0.0172 (7)	0.0156 (8)	0.0123 (7)	0.0039 (6)	0.0060 (6)	0.0012 (6)
C9	0.0221 (8)	0.0232 (9)	0.0137 (7)	0.0056 (6)	0.0089 (7)	0.0001 (6)
C10	0.0248 (8)	0.0222 (9)	0.0141 (7)	0.0068 (7)	0.0071 (7)	−0.0038 (6)
C11	0.0232 (8)	0.0153 (8)	0.0187 (8)	0.0014 (6)	0.0064 (7)	−0.0045 (6)
C12	0.0209 (8)	0.0150 (8)	0.0172 (8)	0.0014 (6)	0.0086 (7)	0.0000 (6)
C13	0.0160 (7)	0.0138 (7)	0.0158 (7)	−0.0031 (6)	0.0093 (6)	−0.0033 (6)
C14	0.0137 (7)	0.0126 (7)	0.0158 (7)	−0.0004 (6)	0.0081 (6)	−0.0001 (6)
C15	0.0148 (7)	0.0181 (8)	0.0141 (7)	−0.0004 (6)	0.0075 (6)	−0.0011 (6)
C16	0.0186 (8)	0.0147 (8)	0.0179 (8)	−0.0025 (6)	0.0097 (6)	−0.0042 (6)
C17	0.0163 (7)	0.0121 (7)	0.0202 (8)	−0.0024 (6)	0.0101 (6)	−0.0002 (6)
C18	0.0367 (10)	0.0200 (8)	0.0252 (9)	0.0000 (7)	0.0235 (8)	0.0021 (7)
C19	0.0539 (12)	0.0235 (9)	0.0157 (8)	−0.0099 (8)	0.0179 (8)	−0.0045 (7)
C20	0.0452 (11)	0.0200 (9)	0.0190 (8)	0.0022 (8)	0.0096 (8)	0.0048 (7)

### Geometric parameters (Å, º)

**Table:**

S1—O1	1.4291 (11)	C8—C9	1.397 (2)
S1—O2	1.4327 (11)	C9—C10	1.381 (2)
S1—N1	1.6554 (13)	C9—H9	0.9300
S1—C1	1.7542 (15)	C10—C11	1.397 (3)
O3—C17	1.3608 (19)	C10—H10	0.9300
O3—C20	1.430 (2)	C11—C12	1.385 (2)
O4—C13	1.3543 (18)	C11—H11	0.9300
O4—C19	1.436 (2)	C12—H12	0.9300
N1—C14	1.425 (2)	C13—C14	1.399 (2)
N1—H1	0.80 (2)	C14—C15	1.382 (2)
N2—C17	1.325 (2)	C15—C16	1.382 (2)
N2—C13	1.329 (2)	C15—H15	0.9300
N3—C4	1.3770 (19)	C16—C17	1.390 (2)
N3—C8	1.390 (2)	C16—H16	0.9300
N3—C18	1.455 (2)	C18—H18A	0.9600
C1—C6	1.388 (2)	C18—H18B	0.9600
C1—C2	1.397 (2)	C18—H18C	0.9600
C2—C3	1.380 (2)	C18—H18D	0.9600
C2—H2	0.9300	C18—H18E	0.9600
C3—C4	1.394 (2)	C18—H18F	0.9600
C3—H3	0.9300	C19—H19A	0.9600
C4—C5	1.416 (2)	C19—H19B	0.9600
C5—C6	1.386 (2)	C19—H19C	0.9600
C5—C7	1.449 (2)	C20—H20A	0.9600
C6—H6	0.9300	C20—H20B	0.9600
C7—C12	1.390 (2)	C20—H20C	0.9600
C7—C8	1.407 (2)		
			
O1—S1—O2	120.41 (6)	C7—C12—H12	120.8
O1—S1—N1	106.10 (7)	N2—C13—O4	119.17 (13)
O2—S1—N1	105.43 (7)	N2—C13—C14	123.64 (14)
O1—S1—C1	109.15 (7)	O4—C13—C14	117.16 (13)
O2—S1—C1	108.71 (7)	C15—C14—C13	117.10 (14)
N1—S1—C1	106.10 (7)	C15—C14—N1	120.55 (13)
C17—O3—C20	116.23 (13)	C13—C14—N1	122.34 (13)
C13—O4—C19	116.68 (12)	C14—C15—C16	120.50 (14)
C14—N1—S1	117.67 (10)	C14—C15—H15	119.7
C14—N1—H1	116.4 (14)	C16—C15—H15	119.7
S1—N1—H1	110.9 (13)	C15—C16—C17	116.98 (14)
C17—N2—C13	117.44 (13)	C15—C16—H16	121.5
C4—N3—C8	108.21 (13)	C17—C16—H16	121.5
C4—N3—C18	125.79 (13)	N2—C17—O3	118.84 (14)
C8—N3—C18	125.74 (13)	N2—C17—C16	124.31 (14)
C6—C1—C2	122.25 (14)	O3—C17—C16	116.85 (14)
C6—C1—S1	119.24 (11)	N3—C18—H18A	109.5
C2—C1—S1	118.31 (11)	N3—C18—H18B	109.5
C3—C2—C1	120.44 (14)	H18A—C18—H18B	109.5
C3—C2—H2	119.8	N3—C18—H18C	109.5
C1—C2—H2	119.8	H18A—C18—H18C	109.5
C2—C3—C4	117.92 (14)	H18B—C18—H18C	109.5
C2—C3—H3	121.0	N3—C18—H18D	109.5
C4—C3—H3	121.0	H18A—C18—H18D	141.1
N3—C4—C3	128.88 (14)	H18B—C18—H18D	56.3
N3—C4—C5	109.52 (13)	H18C—C18—H18D	56.3
C3—C4—C5	121.59 (14)	N3—C18—H18E	109.5
C6—C5—C4	119.85 (13)	H18A—C18—H18E	56.3
C6—C5—C7	133.80 (14)	H18B—C18—H18E	141.1
C4—C5—C7	106.34 (13)	H18C—C18—H18E	56.3
C5—C6—C1	117.89 (14)	H18D—C18—H18E	109.5
C5—C6—H6	121.1	N3—C18—H18F	109.5
C1—C6—H6	121.1	H18A—C18—H18F	56.3
C12—C7—C8	120.01 (14)	H18B—C18—H18F	56.3
C12—C7—C5	133.71 (15)	H18C—C18—H18F	141.1
C8—C7—C5	106.28 (13)	H18D—C18—H18F	109.5
N3—C8—C9	128.59 (15)	H18E—C18—H18F	109.5
N3—C8—C7	109.63 (13)	O4—C19—H19A	109.5
C9—C8—C7	121.78 (15)	O4—C19—H19B	109.5
C10—C9—C8	117.10 (15)	H19A—C19—H19B	109.5
C10—C9—H9	121.5	O4—C19—H19C	109.5
C8—C9—H9	121.5	H19A—C19—H19C	109.5
C9—C10—C11	121.62 (15)	H19B—C19—H19C	109.5
C9—C10—H10	119.2	O3—C20—H20A	109.5
C11—C10—H10	119.2	O3—C20—H20B	109.5
C12—C11—C10	121.16 (16)	H20A—C20—H20B	109.5
C12—C11—H11	119.4	O3—C20—H20C	109.5
C10—C11—H11	119.4	H20A—C20—H20C	109.5
C11—C12—C7	118.33 (15)	H20B—C20—H20C	109.5
C11—C12—H12	120.8		
			
O1—S1—N1—C14	−41.52 (12)	C4—N3—C8—C7	−0.40 (16)
O2—S1—N1—C14	−170.29 (11)	C18—N3—C8—C7	−174.84 (14)
C1—S1—N1—C14	74.49 (12)	C12—C7—C8—N3	−178.80 (13)
O1—S1—C1—C6	−159.85 (11)	C5—C7—C8—N3	1.12 (16)
O2—S1—C1—C6	−26.75 (14)	C12—C7—C8—C9	1.1 (2)
N1—S1—C1—C6	86.21 (13)	C5—C7—C8—C9	−178.95 (13)
O1—S1—C1—C2	25.18 (14)	N3—C8—C9—C10	179.09 (15)
O2—S1—C1—C2	158.28 (11)	C7—C8—C9—C10	−0.8 (2)
N1—S1—C1—C2	−88.76 (13)	C8—C9—C10—C11	−0.2 (2)
C6—C1—C2—C3	1.7 (2)	C9—C10—C11—C12	1.0 (2)
S1—C1—C2—C3	176.48 (11)	C10—C11—C12—C7	−0.7 (2)
C1—C2—C3—C4	−0.5 (2)	C8—C7—C12—C11	−0.3 (2)
C8—N3—C4—C3	178.69 (15)	C5—C7—C12—C11	179.77 (16)
C18—N3—C4—C3	−6.9 (3)	C17—N2—C13—O4	−179.41 (13)
C8—N3—C4—C5	−0.51 (16)	C17—N2—C13—C14	−1.3 (2)
C18—N3—C4—C5	173.93 (14)	C19—O4—C13—N2	−1.0 (2)
C2—C3—C4—N3	179.15 (14)	C19—O4—C13—C14	−179.21 (15)
C2—C3—C4—C5	−1.7 (2)	N2—C13—C14—C15	1.4 (2)
N3—C4—C5—C6	−177.87 (13)	O4—C13—C14—C15	179.54 (13)
C3—C4—C5—C6	2.9 (2)	N2—C13—C14—N1	−177.34 (13)
N3—C4—C5—C7	1.19 (16)	O4—C13—C14—N1	0.8 (2)
C3—C4—C5—C7	−178.08 (13)	S1—N1—C14—C15	76.61 (16)
C4—C5—C6—C1	−1.7 (2)	S1—N1—C14—C13	−104.69 (15)
C7—C5—C6—C1	179.60 (15)	C13—C14—C15—C16	0.2 (2)
C2—C1—C6—C5	−0.5 (2)	N1—C14—C15—C16	178.92 (13)
S1—C1—C6—C5	−175.32 (11)	C14—C15—C16—C17	−1.6 (2)
C6—C5—C7—C12	−2.6 (3)	C13—N2—C17—O3	179.01 (13)
C4—C5—C7—C12	178.51 (16)	C13—N2—C17—C16	−0.4 (2)
C6—C5—C7—C8	177.48 (16)	C20—O3—C17—N2	−5.0 (2)
C4—C5—C7—C8	−1.39 (16)	C20—O3—C17—C16	174.38 (15)
C4—N3—C8—C9	179.67 (15)	C15—C16—C17—N2	1.8 (2)
C18—N3—C8—C9	5.2 (2)	C15—C16—C17—O3	−177.58 (13)

### Hydrogen-bond geometry (Å, º)

**Table:**

*D*—H···*A*	*D*—H	H···*A*	*D*···*A*	*D*—H···*A*
N1—H1···O3^i^	0.81 (2)	2.56 (2)	3.3387 (18)	163 (2)
C18—H18*A*···O1^ii^	0.96	2.45	3.398 (2)	170
C10—H10···O2^iii^	0.93	2.56	3.4887 (19)	177

Symmetry codes: (i) *x*, *y*+1, *z*; (ii) *x*, −*y*+1/2, *z*+1/2; (iii) *x*, −*y*+3/2, *z*+1/2.
